# Intestinal parasitic infections and associated factors in children of three rural schools in Colombia. A cross-sectional study

**DOI:** 10.1371/journal.pone.0218681

**Published:** 2019-07-10

**Authors:** Paula C. Hernández, Liliana Morales, Jacqueline Chaparro-Olaya, Diana Sarmiento, Juan Felipe Jaramillo, Gustavo A. Ordoñez, Fabian Cortés, Lizeth K. Sánchez

**Affiliations:** 1 Laboratorio de Parasitología Molecular, Vicerrectoría de Investigaciones, Universidad El Bosque, Bogotá, Colombia; 2 Instituto de Salud y Ambiente, Vicerrectoría de Investigaciones, Universidad El Bosque, Bogotá, Colombia; 3 Vicerrectoría de Investigaciones, Universidad El Bosque, Bogotá, Colombia; 4 Especialización en Pediatría, Facultad de Medicina, Universidad El Bosque, Bogotá, Colombia; NIH, UNITED STATES

## Abstract

Rural children are one of the populations that are most vulnerable to gastrointestinal parasite infections. Such diseases decrease the quality of life and result in growth and cognitive delays in the long term. This cross-sectional study was conducted to determine the frequency of intestinal parasite infections among rural schoolchildren in the municipality of Apulo, Colombia. A total of 97 stool samples from children aged between 5 and 15 years were collected and examined via direct light microscopy. Microscopic examination was repeated with sediments obtained using a fecal parasite concentrator, and the Kato–Katz test was performed. Frequency of intestinal parasite infection was 100%. *Endolimax nana* (77.35%), *Blastocystis sp*. (71.1%), *Giardia intestinalis* (39.1%), *Entamoeba coli* (25.7%), and the *Entamoeba histolytica/dispar/moshkovskii* complex (9.2%) were the most prevalent protozoa. *Trichuris trichiura* was the most prevalent helminth (12.3%), followed by *Enterobius vermicularis* (6.15%) and *Ascaris lumbricoides* (5.1%). Among the analyzed associated factors, consumption of untreated water increased the risk of acquiring pathogenic intestinal parasites. Finally, because *G*. *intestinalis* was the most prevalent pathogenic protozoan, molecular analysis was conducted to establish genetic assemblages and subassemblages of *Giardia* through sequence-based genotyping of the glutamate dehydrogenase, triose phosphate isomerase, and beta-giardin genes. A total of 14 *G*. *intestinalis*-positive samples were genotyped, which revealed the presence of subassemblages AI (n = 1), AII (n = 7), BIII (n = 2), BIV (n = 2), and BIII/BIV (n = 1) as well as a mixed subassemblage AII + BIII (n = 1). Our results indicate that gastrointestinal parasite infections in the tested population were mainly caused by suboptimal water quality. Moreover, molecular typing of *G*. *intestinalis* suggested contamination of water by animal- and human-derived cysts.

## Introduction

Intestinal parasites are highly prevalent worldwide, particularly in low-income regions, such as most African, Southeast Asian, and Latin American countries [1]. In the latter region, 20%–30% of the inhabitants, specifically those younger than 15 years, develop recurrent parasitosis. Intestinal parasites represent an overlooked health issue in Latin America, perhaps because they are not considered a component of the unfavorable conditions that perpetuate the cycle of poverty [2]. In fact, over the long term, diseases caused by these intestinal parasites can lead to diminished learning capacity in childhood and reduced economic productivity in adulthood [3,4].

The most frequent etiological agents of gastrointestinal parasitosis are waterborne microbes. Consequently, special attention has been paid to water security, as characterized by the World Health Organization strategy WASH: water, sanitation and hygiene [5]. However, low socioeconomic status, uneducated parents, living in crowded houses with insufficient indoor spaces, and contact with pets are factors that increase the incidence of intestinal parasite infections in children [6,7].

Intestinal parasitic diseases affect more than 1 billion people in marginalized and poor communities globally [8], and these diseases include infections by protozoa or helminths. According to the most recent National Survey of Intestinal Parasitism (NSIP) in Colombia, *Trichuris trichiura* (18.4%) and *Ascaris lumbricoides* (11.3%) were the most prevalent helminths in school-age population [9]. Simultaneously, the most prevalent pathogenic protozoa were *Blastocystis sp*. (57.7%) and *Giardia intestinalis* (15.4%). Although the frequency of the *Entamoeba histolytica/dispar/moshkovskii* complex was high (17%), the microscopic methods used by NSIP were not adequate to differentiate between *Entamoeba histolytica* as a pathogenic species and *Entamoeba dispar* or *Entamoeba moshkovskii* as a non-pathogenic species.

Despite its high prevalence, the pathogenicity of *Blastocystis* remains controversial because some studies have associated its presence or absence with certain diseases, whereas others have demonstrated its higher prevalence in healthy controls [10–12]. Therefore, the clinical importance of this parasite remains unclear. On the contrary, *G*. *intestinalis* infections (giardiasis) represent a serious but underestimated public health problem, affecting nearly 200 million people worldwide. Although giardiasis can be asymptomatic, it can also present as an acute disorder (diarrhea and gastrointestinal or general malaise) or as a chronic syndrome. In children, infections can be severe and chronic, affecting their growth and development [13].

Molecular diagnosis of *G*. *intestinalis* allows for the analysis of genetic variability and classification of parasites within eight genotypes or assemblages: A, B, C, D, E, F, G, and H. [14,15]. Within these groups, only *Giardia* assemblages A and B infect humans as well as several animals, whereas the other six assemblages infect a wide range of hosts but not humans. The association between genotypes and clinical characteristics remains debatable; for instance, some researchers have concluded that asymptomatic cases were caused by *G*. *intestinalis* genotype B [16], whereas others have reported the association of this genotype with recurrent diarrhea [17]. Therefore, further studies are warranted to elucidate or reject these possible associations.

This research examined the frequency of intestinal parasite infections and their possible associations with certain socioeconomic factors among schoolchildren in three rural schools in Apulo in the Department of Cundinamarca. Additionally, given the lack of specific epidemiological data pertaining to genetic grouping of *Giardia* populations circulating in Colombia, the other objective was to identify genetic groups of pathogenic parasites in children in these communities.

## Materials and methods

### Ethical considerations

The study was performed with strict adherence to principles in the Declaration of Helsinki and guidelines established by the Ministry of Health of Colombia for procedures involving human subjects. The ethics committee of Universidad El Bosque approved this study (approval #007–2017 issued on April 6, 2017). This study was minimal risk. Approval was obtained from teachers in the selected schools as well as the parents or guardians of each child. Meetings with parents and children were held to explain the purpose and protocol of the study. Signed consent was obtained from the participants’ parents or guardians before initiating stool sample collection, and informed assent was obtained from children who were aged 7–14 years. Following stool analysis, all children infected with pathogenic parasites were treated with secnidazole for protozoan infections and pyrantel pamoate for helminth infections. Treatment was prescribed by a physician according to the infectious diseases treatment guide of PAHO (2004) [18].

### Sociodemographic survey

A questionnaire was completed by interviewing each child’s parents or guardians to obtain socioeconomic and demographic data as well as information on symptoms, environmental factors, and personal hygiene practices (S1 File). Each child underwent a complete clinical examination by a physician associated with the project. Children’s height and weight were recorded using standard calibrated instruments. Z scores for weight-for-age (W/A), height-for-age (H/A), and body mass index-for-age (BMI/A) were calculated using WHO AnthroPlus for personal computers (2009) [19].

### Study population

This cross-sectional study was conducted in Apulo, a municipality located in the southwest of the Department of Cundinamarca at 101 km from Bogotá, the capital of Colombia. Apulo is located between 4°31′15′ N and 74°35′55′ W, at 420 meters above mean sea level. The average temperature in this region is 28°C, and there are 7,477 inhabitants.

The study population comprised children between 5 and 15 years of age, who attended three rural schools of Apulo on 2017. The schools were selected at convenience based on their geographical proximity: Naranjalito, Naranjal, and Pantanos. The sample size was calculated using the approach proposed by Khan and Sempos [20] to estimate the incidence and frequency of parasitosis. A total of 97 children were enrolled, of which 57 children attended Naranjalito, 9 attended Naranjal, and 31 attended Pantanos.

### Sample collection and parasitological analyses

Capped containers with wide mouths were provided to each participant with detailed instructions regarding stool collection. Stool samples (one for each child) were collected in the early morning from participants at their schools. Immediately after collection, all specimens were labeled with a code assigned to each participant, written in a combination of a letter (“P” for samples from Pantanos, “T” for samples from Naranjalito and “N” for samples from Naranjal) and a consecutive number in order of collection. Samples were transported in cold containers to the Apulo public health center for microscopic stool examination.

All stool samples were examined macroscopically, and their characteristics were recorded. Slides were prepared directly by wet mounting in saline and iodine solutions and observed via direct light microscopy. For each sample, sediment was obtained using a Mini Parasep SF fecal parasite concentrator (DiaSys Ltd, Berkshire, England), and slides were prepared with saline or iodine solution to repeat microscopic examination. The remained concentrated was stored at −20°C without preservatives for DNA extraction. Finally, each sample was processed using a Kato–Katz kit (Sterlitech Corp., USA). All examinations were repeated two times by two experienced microscopists.

### *Giardia* genotyping and phylogenetic analysis

DNA was extracted from concentrated samples (n = 97) using the QIAamp DNA Stool Mini Kit (Qiagen, Germany) according to the manufacturer’s instructions with minor modifications. First, 300 μL of concentrated sample were combined with lysis buffer as usual, but adding glass beads and 150 μL of a 10% polyvinylpyrrolidone solution. In addition, incubation time and temperature were increased (from 5 min and 70°C to 30 min and 95°C) and glycogen was added during ethanol precipitation [21]. DNA was eluted in 100 μL elution buffer (Qiagen) and stored at −20°C until use.

To identify *G*. *intestinalis* assemblages, three gene loci were used: glutamate dehydrogenase (*gdh*), beta-giardin (*bg*), and triose phosphate isomerase (*tpi*). Semi-nested polymerase chain reaction (PCR) was performed to amplify a *gdh* gene fragment (~432 bp), as described by Read et al. [22]. PCR was performed using primer pairs GDHeF/GDHiR (0.5 μM each) in the primary reaction and GDHiF/GDHiR (0.5 μM each) in the secondary reaction as well as 1.5 mM MgCl_2_. The cycling conditions were 10 min at 94°C; 35 cycles at 94°C for 35 s, 61°C for 35 s, and 72°C for 50 s; and final extension at 72°C for 7 min. Amplification of the *bg* and *tpi* loci was performed using nested PCR as described by Lalle et al. [23] and Sulaiman et al. [24], respectively. For *bg* gene amplification (~511 bp), PCR was performed using primers pairs G7/G759 (0.4 μM each) in the primary reaction and GiarF/GiarR (0.4 μM each) in the secondary reaction as well as 1.5 mM MgCl_2_. The cycling conditions for primary PCR comprised an initial step at 94°C for 7 min, followed by 35 cycles at 94°C for 30 s, 65°C for 30 s, and 72°C for 1 min and final extension at 72°C for 7 min. The same conditions were used for secondary PCR except that the annealing temperature was 55°C. For *tpi* gene amplification (~530 bp), PCR was performed using primers pairs AL3543/AL3546 (0.2 μM each) in the primary reaction and AL3544/AL3545 (0.2 μM each) in the secondary reaction as well as 3 mM MgCl_2_. The cycling conditions for the primary and secondary PCR comprised an initial step at 94°C for 5 min, followed by 35 cycles at 94°C for 45 s, 50°C for 45 s, and 72°C for 1 min and final extension at 72°C for 10 min.

All PCRs were performed with a final volume of 25 μL containing 0.5 U GoTaq DNA polymerase (Promega), 200 μM dNTPs, and 5 μL extracted DNA (for primary PCR). For secondary PCR, 1 μL of the primary PCR product was used as template. PCR was performed using T100 Thermal Cycler (BIORAD). DNA from G. *intestinalis* strains WBC6 for subassemblage AI, Bris-136 for subassemblage AII, and Ad-28 for subassemblage BIV was used as positive controls.

Secondary PCR products were purified, and both strands were sequenced (MACROGEN, Korea). The obtained chromatograms were analyzed and edited, and sequences were aligned using MEGA Version 7.0 [25]. Reference sequences, retrieved from GenBank, representing each assemblage (S1 Table) were employed for phylogenetic analyses using neighbor-joining and maximum likelihood algorithms for our sequences of the three loci. The Tamura–Nei parameter model was used to construct phylogenetic trees with the highest likelihood logarithm value. Reliability of clusters was evaluated using a bootstrap of 1000 iterations. Novel sequences obtained in this study were deposited in GenBank under accession numbers MK026072–MK026080, MK035579–MK035597, MK907782, MK919467 and MK919468.

### Statistical analysis

Descriptive statistics, including mean, median, standard deviation (SD), and interquartile ranges, were used to characterize the study population according to the assumption of normality, as evaluated using the Shapiro–Wilk test. Group comparisons (children with and without intestinal parasite infections) of demographic and other characteristics were performed using Z-test or Wilcoxon’s rank sum test for continuous variables as appropriate. For comparing proportions or categorical variables, Z-proportion, chi-squared, or Fisher’s exact test was used as appropriate. The association between the analyzed factors and presence of intestinal pathogenic parasites was estimated using crudes odds ratios (ORs) as well as multivariated ones calculated by a non-conditional logistic regression, then a chi-squared test was performed. Interacting and confounding variables were selected through stepwise regression using a probability to enter of 0.1 and a probability to remove of 0.25. All logistic models were evaluated using the Hosmer–Lemeshov goodness-of-fit test. A p ≤ 0.05 was considered statistically significant. Statistical analyses were performed using STATA V.14 (Stata Corporation, College Station, TX, USA).

## Results

### Frequency of intestinal parasitosis

A total of 97 children were included in the study. The mean age of participants was 9.46 ± 2.62 years, and distribution by sex was uniform. The overall frequency of intestinal parasite infections was 100% among the included rural schoolchildren. Children were divided into two groups based on the presence (PP; pathogenic protozoa + helminths) or absence (NPP; non-pathogenic protozoa) of intestinal pathogenic parasites. Frequency of PP was 46.4% (n = 45), with a slight female predominance, albeit without statistical significance (n = 24, 53.3%). Regarding anthropometric measurements, 2 (2.1%), 7 (7.2%), and 5 (5.1%) children showed Z scores lower than −2 SD for W/A, H/A, and BMI/A, respectively. No difference was found in Z scores for W/A, H/A, and BMI/A between children with PP and NPP (Table 1).

**Table 1:** Demographic characteristics of population with pathogenic parasites (PP) and without pathogenic parasites (NPP).

Variable	Total population (n = 97)	PP (n = 45)	NPP (n = 52)	p value
**Age (years)**				
Mean (SD)	9.46 (+/- 2.62)	9.80(+/-2.59)	9.17 (+/-2.63)	0.240*
**Sex**				
Males (n—%)	49–50.52	21–46.67	28–53.85	0.480**
**W/A (Z score)†**				
Mean (SD)	-0.24 (+/-1.06)	-0.26 (1.01)	-0.22 (1.11)	0.901*
**H/A (Z score)**				
Mean (SD)	-0.60 (+/-0.93)	-0.61 (0.97)	-0.58 (+/-0.90)	0.890*
**BMI/A (Z score)**				
Median	0.01	0.14	0.0	0.599‡
IR	-0.64, 0.77	-0.53, 0.87	-0.75, 0.75	

BMI: Body Mass Index; SD: Standard Deviation IR: interquartile range; W: weight; M: mass; H: Height; A: Age.

* Z test for difference between two means

** Z test for difference between two proportions

† This variable was estimated only for children ≤10 years old (n = 58).

‡ Wilcoxon Rank-sum test.

Distribution of intestinal parasites in schoolchildren is shown in Table 2. Overall, nine intestinal parasites were identified: *Iodamoeba butschlii* (2.06%), *A*. *lumbricoides* (5.15%), *Enterobius vermicularis* (6.19%), *Entamoeba histolytica/dispar/moshkovskii* complex (9.28%), *T*. *trichiura* (12.37%), *Entamoeba coli* (25.77%), *G*. *intestinalis* (39.18%), *Blastocystis sp*. (71.13%), and *Endolimax nana* (77.32%). *Giardia intestinalis* (the most prevalent pathogenic protozoan) and *T*. *trichiura* (the most prevalent helminth) infections were significantly more prevalent in children attending Naranjalito (*p* = 0.040 and *p* = 0.015, respectively). Double infections were the most prevalent (38%), followed by triple infections (2726.8%). Single and multiple infections were found in 17.53% of the children. The combination of *Blastocystis sp*. and *Endolimax nana* was the most frequent double infection, affecting 26% of the children. The combination of *Blastocystis sp*., *Endolimax nana*, *T*. *trichiura*, *A*. *lumbricoides*, *Entamoeba coli*, and the *Entamoeba histolytica/dispar/moshkovskii* complex was detected in a child attending Naranjalito.

**Table 2:** Distribution of intestinal parasitic infections by school.

Intestinal parasites		n	%	Naranjal (n = 9)	Pantanos (n = 31)	Naranjalito (n = 57)	p value*
				n—%	n—%	n—%	
**Pathogenic protozoa**	*Giardia intestinalis*	38	39.18	1–11.11	9–29.03	28–49.12	**0.040**
	*Entamoeba his/dis/mos*	9	9.28	0–0.0	3–9.68	6–10.53	0.881
**Non-pathogenic protozoa**	*Endolimax nana*	75	77.32	8–88.89	25–80.65	42–73.68	0.623
	*Blastocystis sp*.	69	71.13	7–77.78	22–70.97	40–70.18	1.000
	*Entamoeba coli*	25	25.77	3–33.33	14–24.56	8–25.81	0.829
	*Iodamoeba butschlii*	2	2.06	0–0.0	1–3.25	1–1.75	1.000
**Helminths**	*Ascaris lumbricoides*	5	5.15	0–0.0	0–0.0	5–8.77	0.257
	*Enterobius vermicularis*	6	6.19	0–0.0	0–0.0	6–10.53	0.160
	*Trichuris trichiura*	12	12.37	1–11.11	0–0.00	11–19.30	**0.015**
Single infections		17	17.53	2–22.22	8–25.81	7–12.28	
Double infections		37	38.14	4–44.44	11–35.48	22–38.60	
Triple infections		26	26.80	2–22.22	10–32.26	14–24.56	
Quadruple, fivefold and sixfold infections		17	17.53	1–11.11	2–6.45	14–24.56	

*p-value calculated using Fisher exact-test.

Bold numbers: statistically significant.

### Sociodemographic characteristics of children with pathogenic parasites (PP) and non-pathogenic protozoa (NPP)

Statistically significant differences were identified among the schools in terms of the presence of PP or NPP according to the sociodemographic characteristics of children (Table 3). Naranjalito showed the highest frequency of PP, whereas Naranjal showed the lowest frequency (p = 0.021). Among the lifestyle conditions of the children’s families, students who lived in crowded homes (six or more members in a single household) were significantly more likely to be infected with PP (p = 0.023), and consumption of untreated water increased the risk of acquiring PP (p = 0.001). There was no significant difference between the two groups in terms of family income, parents’ educational level, house construction material, use of shoes, consumption of raw vegetables or fruits, washing vegetables or fruits before consumption, presence of gastrointestinal symptoms, and history of intestinal parasite infections (Table 3).

**Table 3:** Sociodemographic characteristics, personal hygiene practices, and symptoms of children with intestinal Pathogenic Parasites (PP) and Non-Pathogenic Parasites (NPP).

Characteristic	Total population (n = 97)	PP (n = 45)	NPP (n = 52)	p value
**School, n (%)**				
Naranjal	9 (9.28)	2 (4.44)	7 (13.46)	**0.021**^a^
Pantanos	31 (31.96)	10 (22.22)	21 (40.38)	
Naranjalito	57 (58.76)	33 (73.33)	24 (46.15)	
^e^**Family income, n (%)**				
<500,000 COP	73 (76.84)	37 (86.05)	36 (69.23)	0.053^a^
≥500,000 COP	22 (23.16)	6 (13.95)	16 (30.77)	
^e^**Educational level of parents, n (%)**				
Technical	7 (7.37)	3 (6.98)	4 (7.69)	0.874^a^
High school	48 (50.53)	21 (48.84)	27 (51.92)	
Primary	39 (41.05)	18 (41.86)	21 (40.38)	
^e^**Family size (number of residents per household)**				
Median	5	6	5	**0.023**^c^
IR	4–7	4–7	4–6	
**House construction material, n (%)**				
Cement/brick	83 (87.37)	39 (90.70)	44 (84.62)	0.297^a^
Adobe/giant bamboo	9 (9.47)	2 (4.65)	7 (13.46)	
Other	3 (3.16)	2 (4.65)	1 (1.92)	
**Treatment of water for consumption, n (%)**				
Boiled/chlorinated/filtrated	72 (74.23)	26 (57.78)	46 (88.46)	**0.001**^d^
None	25 (25.77)	19 (42.22)	6 (11.54)	
**Use of shoes, n (%)**				
No	77 (81.05)	37 (86.05)	40 (76.92)	0.259^b^
**Raw fruit and vegetable consumption, n (%)**				
Yes	74 (77.89)	35 (81.40)	39 (75.00)	0.455^b^
**Washing fruits and vegetables before consumption, n (%)**				
Yes	92 (96.84)	40 (93.02)	52 (100.00)	0.053^b^
**History of intestinal parasite infection, n (%)**				
Yes	62 (63.92)	30 (66.67)	32 (61.54)	0.600^b^
**History of diarrhea in the past 15 days, n (%)**				
Yes	13 (13.40)	4 (8.86)	9 (17.31)	0.225^b^
**History of abdominal pain in the past 15 days, n (%)**				
Yes	49 (50.52)	21 (46.67)	28 (53.85)	0.481^b^
**Members of the same household having diarrhea for the past 15 days, n (%)**				
Yes	16 (16.49)	6 (13.33)	10 (19.23)	0.435^b^
**Abdominal pain at the time of physical examination, n (%)**				
Yes	19 (19.59)	9 (20.0)	10 (19.23)	0.924^b^

IR: interquartile range; 3000 Colombian pesos (COP) = 1 USD.

^a^Fisher’s exact test.

^b^Z-test for difference between two proportions.

^c^Wilcoxon’s rank sum test.

^d^Pearson’s chi-squared test.

^e^These data were available for only 95 children.

Bold numbers: statistically significant

### Factors associated with the presence of PP and NPP

The crude model showed that children living in houses with more than seven residents had a 3.38-fold higher probability of being infected with PP than those living with four or fewer family members; however, this association did not remain significant in the multivariate logistic regression model (Table 4). The risk of infection was 5.6-fold higher in children whose families consumed untreated water than in children whose families consumed treated water in both the crude and multivariate logistic regression models (OR = 3.69; 95% confidence interval = 1.11–12.20) (*p* < 0.05) (Table 4).

**Table 4:** Associated factors with intestinal infections in children of three rural schools in Colombia with intestinal Pathogenic Parasites (PP) and Non-Pathogenic Parasites (NPP).

Factor	PP n = 45		NPP n = 52		Crude model			Multivariate model**		
	%	n	%	n	OR*	IC 95%	*p*	OR**	IC 95%	*p*
**Family size**										
≤ 4	13	28.89	18	34.62	1.0			1.0		
5–6	10	22.22	25	48.08	0.55	0.19–1.54	0.258	1.15	0.34–3.87	0.817
≥ 7	22	48.89	9	17.31	3.38	1.17–9.78	**0.023**	2.44	0.75–7.94	0.137
**Treatment of water for consumption**										
Boil/chlorinated/filtrated	26	57.78	46	88.46	1.0			1.0		
None	19	42.22	6	11.54	5.60	1.98–15.79	**0.001**	3.69	1.11–12.20	**0.032**
**Use of shoes**										
No	37	86.07	40	76.92	1.0			1.0		
Yes	6	13.96	12	23.08	0.54	0.18–1.58	0.263	0.66	0.17–2.54	0.555
**Raw fruit and vegetable consumption**										
No	8	18.60	13	25.0	1.0			1.0		
Yes	35	81.40	39	75.0	1.45	0.54–3.93	0.456	1.46	0.45–4.66	0.519
**History of intestinal parasites infections**										
No	15	33.33	20	38.46	1.0			1.0		
Yes	30	66.67	32	61.54	1.25	0.54–2.87	0.600	1.24	0.42–3.61	0.689
**History of diarrhea the last 15 days**										
No	41	91.11	43	82.69	1.0			1.0		
Yes	4	8.89	9	17.31	0.46	0.13–1.63	0.233	0.78	0.18–3.28	0.738
**History of abdominal pain the last 15 days**										
No	24	55.33	24	46.15	1.0			1.0		
Yes	21	46.67	28	53.85	0.75	0.33–1.66	0.481	0.81	0.30–2.16	0.682
**Members of the same household with diarrhea the last 15 days**										
No	39	86.67	42	80.77	1.0			1.0		
Yes	6	13.33	10	19.23	0.64	0.21–1.94	0.437	0.62	0.17–2.25	0.468
**Washing hands before meals**										
Always	22	51.16	25	48.08	1.0			1.0		
Sometimes	20	46.51	24	46.15	0.37	0.03–3.91	0.415	0.71	0.04–11.06	0.808
Never	1	2.33	3	5.77	0.94	0.41–2.16	0.897	0.67	0.23–1.92	0.460
**Washing hands after using toilet**										
Always	39	86.67	46	88.46	1.0			1.0		
Sometimes	4	8.89	5	9.62	2.35	0.2–27.01	0.490	2.45	0.3–26.2	0.442
Never	2	4.44	1	1.92	0.94	0.23–3.75	0.934	0.84	0.15–4.59	0.849
**How hands are washed**										
With water and soap	33	76.74	41	78.85	1.0			1.0		
Just water	10	23.26	11	21.15	1.12	0.42–2.98	0.806	1.52	0.52–4.40	0.434

OR, odds ratio.

*Estimated using logistic regression.

**Logistic regression adjusted by child’s age, school, family income, crowding, material of construction of household and drinking water when the factor studied did not included one of those variables as independent variable.

### Genotyping of *G*. *intestinalis* samples

**Fig 1:**
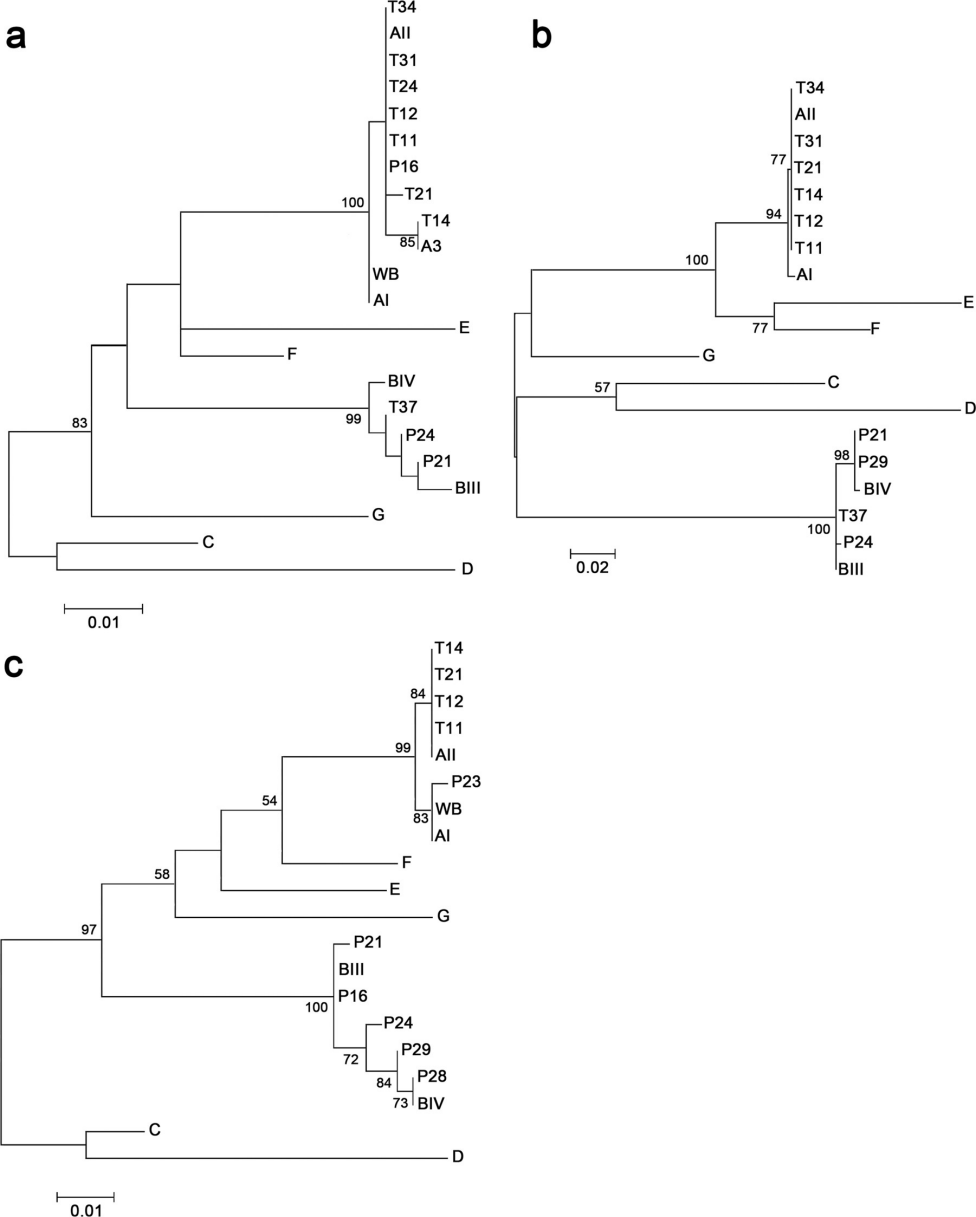
Phylogenetic trees of *Giardia intestinalis* based on nucleotide sequences of three gene loci. Sequences of the *bg* (a), *tpi* (b), and *gdh* (c) genes retrieved from samples obtained in this study were compared with reference sequences from GenBank. Trees were constructed using maximum likelihood analysis based on genetic distances calculated using the Tamura–Nei parameter model implemented in MEGA Version 7.0. Bootstrap values >50% from 1000 interactions are indicated at nodes.

Of the 39 samples positive for *G*. *intestinalis*, 14 were successfully amplified and sequenced using at least one of the three loci: *gdh* (n = 10), *tpi* (n = 10), and *bg* (n = 11). A total of 6 samples were amplified using all three loci, whereas 2 and 5 were amplified using one and two loci, respectively. Fig 1 shows phylogenetic relationships of sequences of the three analyzed loci with reference sequences from National Center for Biotechnology Information. The obtained dendrograms differentiated assemblages and subassemblages with high bootstrap support.

Genotyping results of *G*. *intestinalis* sequences generated using the three loci in the present study corresponding to each sample are shown in Table 5. Sequence analyses revealed that eight and five samples corresponded to assemblages A and B, respectively, and one sample (P16) presented two assemblages (AII and BIII). Multilocus genotyping was obtained for P21, P24, T11, T12, T14 and T21 samples.

**Table 5:** *Giardia* genotyping results of DNA samples obtained from children of three rural schools of Apulo.

*Sample*	*Molecular marker*						*MLG*
	*gdh*^*a*^		*tpi*^*b*^		*b^g^*^*c*^		
	Assem.	Subtype	Assem.	Subtype	Assem.	Subtype	
*P21*	BIII	-	BIV	-	BIII	-	BIII/BIV
P24	BIII	-	BIII	-	B±	-	BIII
T11	AII	A2	AII	A2	AII	A2	AII-1
T12	AII	A2	AII	A2	AII	A2	AII-1
T14	AII	A2	AII	A2	AII	A3	AII-3
T21	AII	A2	AII	A2	AII	New AII	AII-New
P16	BIII	-	*	-	AII	A2	-
P23	AI	New AI	NA	-	NA	-	-
P28	BIV	-	NA	-	NA	-	-
P29	BIV	-	BIV	-	NA	-	-
T24	NA	-	NA	-	AII	A2	-
T31	NA	-	AII	A2	AII	A2	-
T34	NA	-	AII	A2	AII	A2	-
T37	NA	-	BIII	-	BIV	-	-

Assem.: Assemblage.

MLG: Multilocus Genotyping.

NA: non-amplified

±: indicate samples that could not be assigned to a specific sub-assemblage

* Chromatograms had many double peaks.

^*a*^ Fragment size: 369 nt.

^*b*^ Fragment size: 449 nt.

^*c*^ Fragment size: 473 nt.

Sequences sizes were smaller than those expected from PCR products since ends were compromised during chromatograms edition (Table 5).

Regarding samples classified into assemblage A, only one (P23) corresponded to subassemblage AI; this sample harbored a single nucleotide polymorphism (SNP) (G to A at position 235) at the *gdh* locus compared with the reference sequence L40509 (isolate Ad-1) and was named New AI subtype. Regarding sequences classified into subassemblage AII, T11, T12, T14, and T21 exhibited complete sequence identity with the reference sequence AY178737 (isolate Bris-136) at the *gdh* locus. Regarding the *tpi* locus, all sequences (T11, T12, T14, T21, T31, and T34) displayed complete identity with the reference sequence U57897 (isolate JH). Regarding the *bg* locus, six sequences (T11, T12, T21, T31, T34, and P16) were classified into subassemblage AII (compared to the reference sequence AY072723, isolate KC8). Sequence T21 harbored an SNP (A to T at position 573) compared to sequence AY072723, and was named New AII subtype, whereas sequence T14 showed complete sequence identity with subtype A3 (compared to the reference sequence AY072724, isolate ISSGF7) (Fig 1A). In general, samples classified into assemblage A exhibited high sequence homogeneity. Conversely, samples classified into assemblage B showed high genetic diversity at all loci. Sequence analysis of the *gdh* locus revealed that various positions within sequence P16 were heterogeneous (compared to the BIII reference sequence AF069059, isolate BAH-12), as characterized by the presence of two overlapping nucleotide peaks at specific positions (Table 6). There was only one heterogeneous position in samples P21 and P29. Sample P28 exhibited complete sequence identity with assemblage BIV (compared to sequence AY178738). Finally, samples P24 and P29 harbored two and five polymorphic positions, respectively, compared with the BIII reference sequence.

**Table 6:** Polymorphisms and heterogeneous positions at the *gdh*, *bg* and *tpi* loci of assemblage B samples compared to reference sequences obtained from GenBank.

Sample (ref. sequence)	Nucleotide position from start of reference gene															
	***Gdh***															
	**99**	**219**	**231**	**237**	**279**	**300**	**309**	**312**	**315**	**321**	**330**	**351**	**372**	**394**	**402**	**405**
BIII (AF069059^a^)	C	T	T	T	T	T	C	T	T	T	C	C	G	C	G	A
P21	.	.	.	.	.	.	**Y**	.	.	.	.	.	.	.	.	.
P16	.	.	**Y**	**Y**	**Y**	**Y**	.	**K**	**Y**	**Y**	.	.	**R**	**Y**	.	**R**
BIV (AY178738^b^)/P28	T	C	.	C	.	.	.	.	.	.	T	T	.	.	A	.
P29	**Y**	C	.	C	.	.	.	.	.	.	T	.	.	.	A	.
P24	.	.	.	.	.	.	.	.	.	.	T	.	.	.	A	.
	***Bg***															
	**183**	**228**	**309**	**312**	**519**	**564**										
BIII (AY072726^c^)	A	G	C	C	T	C										
P24	G	A	.	T	.	.										
T37	G	A	T	T	.	.										
BIV (AY072728^d^)	G	A	T	T	C	T										
P21	.	A	.	T	.	.										
	***Tpi***															
	**34**	**108**	**111**	**132**	**153**	**372**										
BIII (AF069561^a^)/T37	C	C	C	A	G	G										
P21/P29	T	T	T	.	A	.										
BIV (AF069560^e^)	T	T	T	.	A	A										
P24	.	.	.	G	.	.										

Dots indicate identity to the BIII reference sequence. Heterogeneous positions are indicated in bold.

Standard mixbase definition: **Y**: C/T; **K**: G/T; **R**: A/G.

^a^Isolate BAH-12

^b^Isolate Ad-28

^c^Isolate LD-18

^d^Isolate ISSGF4

^e^Isolate Ad-19

Regarding the *bg* locus, sequenced samples exhibited no ambiguous nucleotides. Samples P24, T37, and P21 showed substitutions at positions 183, 228, 309, and 312 compared with the BIII reference sequence (AY072726, isolate LD18) (Table 6). Samples P24 and T37 had substitutions at positions 183 (A to G), 228 (G to A) and 312 (C to T). Sample T37 had also a substitution at position 309 (C to T). Sample P21 exhibited changes at positions 228 (G to A) and 312 (C to T).

Regarding the *tpi* locus, sample T37 displayed complete sequence identity with the BIII assemblage (sequence AF069561, isolate BAH-12). Samples P21 and P29 exhibited the same polymorphisms as did the BIV reference sequence (AF069560, isolate Ad-19), except position 372 (from G to A). Sample P24 showed only one polymorphism (at position 132) compared with the BIII reference sequence. These sequences exhibited no ambiguous nucleotides. In general, sequences assigned to assemblage B showed certain substitutions at specific positions, rendering the subassemblage discrimination difficult (Table 5).

## Discussion

In this study, the frequency of intestinal parasite infection was 100%, which is higher than the global frequency of 81% reported by NSIP [9], although Apulo was not included in that surveillance. Similar results were obtained in a study of children living in the highlands of Huanuco, Peru, in which the prevalence of intestinal parasite infection was 100% [26]. Surveillances of school-age populations in rural regions of other South American countries have reported lower prevalence of intestinal parasite infection, namely 68% in Chile [27] and 60% in Brazil [28]. Recent studies of schoolchildren residing in rural areas of Lebanon [29], Nigeria [30], and Malaysia [31] have reported respectively 85%, 86%, and 98% prevalence of intestinal parasite infections.

Contrary to the national survey, we considered *Blastocystis sp*. as a non-pathogenic parasite because its potential pathogenicity has not been confirmed. Then, regarding protozoan parasites, *Endolimax nana* was the most prevalent commensal parasite (77.32%), followed by *Blastocystis sp*. (71%), whereas *G*. *intestinalis* was the most prevalent pathogenic parasite (39.18%). Conversely, NSIP has identified *Blastocystis sp*. as the most frequent pathogenic parasite (57.7%). The presence of commensal parasites is an indicator of environmental fecal contamination that is potentially related to poor hygienic–sanitary habits and/or the absence of adequate aqueduct and sewerage systems. These parasites become important as indicators of the ingestion of fecal matter and therefore are a risk factor to acquire other parasitosis [32].

In case of pathogenic protozoa, the frequency of *Entamoeba histolytica/dispar/moshkovskii* complex infection (9.28%) was lower in our study population than that reported in Colombia by NSIP (17.0%). *Giardia intestinalis* was significantly more frequent in children attending Naranjalito (49.12%, Table 2), wherein the highest frequency of PP was found. Regarding soil-transmitted helminths, *T*. *trichiura* was the most prevalent (12.37%), followed by *A*. *lumbricoides* (5.15%). These percentages were lower than those reported in Colombia by NSIP (18.4% and 11%, respectively). The frequency of *Enterobius vermicularis* (6.19%) was particularly high in the studied communities compared with that in Colombia reported by NSIP (1%). This result is particularly worrisome because *Enterobius vermicularis* is the most frequent helminth worldwide, with most infected individuals being asymptomatic.

In this study, monoinfection was less frequent (17.53%). Majority of the children were infected with two or more parasites (83.4%). The frequency of polyparasitism was high in these communities compared with that reported in Colombia by NSIP, in which a low rate of multiple infections was identified. Nevertheless, other studies conducted along the Caribbean Coast of Colombia and in the municipality of Tuta (Boyacá) have recorded comparable rates of polyparasitism with the rate in our study (89.2 and 84%, respectively) [33,34]. Taken together, these results suggest that children live in highly contaminated environments either in their homes or at schools where they spend most of their time [35].

No significant association was identified between age, sex, or anthropometric indices (H/A, W/A, and BMI/A) and infection with PP because all age groups were almost equally exposed. Among other factors associated with infection with PP, including family size, school, and untreated water consumption, the absence of water treatment was the best predictor of PP infection according to multivariate analysis. Low family income was rather common among children with PP infection, although no significant association was identified between the two factors. Low socioeconomic status has been reported as a risk factor for intestinal parasitosis, which is associated with non-access to education, poor sanitary conditions and hygiene, and living in crowded houses and shanty areas [36–38].

Collectively, our findings indicate the existence of different contamination sources and transmission pathways among the surveyed communities. Our statistical analysis demonstrated that consumption of untreated water was the primary cause of such a high prevalence of PP among children in the three schools (OR = 3.69, p = 0.032). The cornerstone of intestinal parasitosis prevention is the use of treated water for drinking and cooking, and our findings are consistent with those of multiples studies in other schools [26,29,30]. Majority of the parents declared consumption of untreated water at home. The schools used rainwater collected via simple systems of capture through the roofs and storage in plastic or cement tanks. Naranjalito and Pantanos obtained water through a pipeline connection from rural community aqueducts, which collect water from different water sources with a periodicity of two or three times a week and daily, respectively. The schools stored water in concrete tanks and distributed it only after screening for coarse particles without any physical or chemical treatment for purification. Naranjal was supplied directly from an unprotected natural source of water. Food handlers at the schools filtered and boiled the water for consumption (*in situ* treatment) except at Naranjalito, where water was only filtered but not boiled. However, treated water was not consistently available throughout the school day, indicating that students also drank untreated water directly from the tap. In this regard, it is essential to investigate further whether differences in microbiological quality of water sources for human consumption explain, at least partially, the observed distribution of PP in the studied schools. This analysis should be conducted by directly sampling the sources of water supply to schools and homes before (storage) and after the *in situ* treatment.

The present study provides molecular data regarding the diversity of *G*. *intestinalis*, which was the most frequent pathogen among schoolchildren in Apulo. We performed sequence typing of the *bg*, *gdh*, and *tpi* genes to characterize *G*. *intestinalis* in the collected samples. The phylogenetic analysis confirmed the monophyletic status of assemblages A and B. Samples from Naranjalito school (T label) were largely similar and exhibited either AI or AII assemblages, except for T37 sample. Conversely, the samples from Pantanos school (P label) largely harbored B assemblages. Sequence analysis revealed that *G*. *intestinalis* assemblages A and B were present in the study population at equal proportions. In another study in La Virgen, Colombia, high frequency of genotype B (90%) was reported in schoolchildren [39]. Interestingly, the Apulo municipality and La Virgen hamlet are geographically close within the Department of Cundinamarca but comparatively our results were different. Similarly, another study along the Caribbean Coast of Colombia has revealed predominance of *G*. *intestinalis* genotype B in children younger than 7 years [40]. A study of indigenous children in the Colombian Amazon region [41] has reported high prevalence of assemblage AI (61%). Another study performed in the central region of Colombia has found an equal distribution of assemblages A and B in children [42]. Based on these findings, the geographical distribution of *G*. *intestinalis* assemblages from human samples lack a spatial structuring across Colombia, which is similar to the global trend. For instance, several studies in Mexico [43–45] had reported only the presence of genotype A (AI and AII) in several regions of the country until recent discoveries of genotype B in humans in southwestern [46] and northwestern regions [47].

Furthermore, our sequence analysis demonstrated that assemblage B samples more frequently exhibited genetic polymorphisms than did assemblage A samples. Most assemblage A samples were successfully characterized at the subassemblage level at the three loci (Table 5). The six samples with complete intra-assemblages for the three genes (*gdh*, *tpi*, and *bg*) were classified as MLG: three for assemblage A, two for assemblage B, and a new MLG combination of assemblage A. Regardless of the gene analyzed, all sequences matched those of previously described samples, except an SNP detected in the *bg* gene in sample T21 (not described before), which lead us to postulate a new MLG. The same for sample P23 for which a not previously reported polymorphism on *gdh* locus (corresponding to assemblage AI) was found. No sequences of assemblage A displayed heterogeneous positions. Most of the assemblage A isolates were assigned to subassemblage AII consistently across the three loci; these findings corroborate results of other previous studies in humans using multilocus sequence typing [48–50], and contrary to what was obtained in the study of the Colombian Amazon region, as previously was described [41].

Molecular analysis of assemblage B samples was complicated by the occurrence of sequences with heterogeneous positions (double peaks) with overlapping nucleotides observed across the three loci; this is consistent with previous reports that assemblage B samples exhibited more subtypes than did assemblage A samples [17,21,48]. In this study, most of the SNPs reported across the three loci produced clear chromatogram readings in the forward and reverse directions, although a number of double peaks were also detected, particularly at the *gdh* locus. P16 sample was simultaneously classified as assemblage BIII and AII based on the *gdh* and *bg* genes analysis, respectively. This result was confirmed by PCR–RFLP (S2 File). The assignment of *Giardia* to different assemblages at different loci in the same isolate has been frequently reported and is known as ‘assemblage swapping’ [17, 48, 51, 52]. There are two explanations for ‘assemblage swapping’ according to Ryan and Caccio [51]: (i) the existence of recombinant isolates in which genetic exchanges have occurred, and (ii) mixed infections, i.e. the presence of genetically different cysts in a fecal sample coupled with preferential amplification of one assemblage at a particular locus and of another assemblage at another locus.

The molecular data presented here show a high level of genetic diversity at the nucleotide level within assemblage B and some polymorphic positions for samples that belong to assemblage A. Our work provides a new insight into the genetic diversity of *Giardia*, improving current understanding of the disease epidemiology by elucidating the dynamics of giardiasis in Colombia. Further studies on molecular epidemiology using MLG are required in local samples in order to provide more evidence on parasite transmission dynamics. It would be very interesting to continue studying these samples to analyze and corroborate the genetic diversity with other more variable markers or with whole genome analysis using next generation sequencing technologies.

Previous studies have assessed the correlation between assemblage and symptoms [16, 53]. However, a single *Giardia* monoinfection was found in this study and that is the only case wherein we could suggest that the observed symptoms were indeed caused by *G*. *intestinalis* and not by other parasites. A statistically significant association (OR = 4.89, p = 0.006) between giardiasis and untreated water consumption (S2 Table) was confirmed via multivariate analysis. Contamination of water by *Giardia* cysts of several origins (animal and human) may explain the diversity of assemblages found in this study.

This study has limitations considering that no parasitological examination of animal feces, water, and soil samples was performed. Consequently, transmission routes of intestinal parasite infections could not be verified, and zoonotic potential of *G*. *intestinalis* could not be ascertained. Multiple interventions should be implemented for schoolchildren, households, and communities to reduce incidence of intestinal parasite infections, such as establishing adequate access to safe drinking water, providing persuasive education on these infections, and improving personal and environmental hygiene habits. Local government authorities must implement preventive strategies as well as controlled and regular mass deworming programs. We believe that the epidemiological data generated in this study may help local and regional health authorities and decision makers to optimize and implement resources to improve the quality of life of people (particularly children) in rural areas and combat intestinal parasite infections. The results of this study have already been communicated to the local authorities.

## Supporting information

S1 Table Accession numbers obtained from Genbank for sequences used for *G*. *intestinalis* assemblage identification. (PDF)

S2 Table Associated factors with *G*. *intestinalis* infection in children of three rural schools in Colombia. (PDF)

S3 Table Alignments of *gdh* sequences of samples and reference sequences for assemblage A and B.
(XLS)

S4 Table Alignments of *bg* sequences of samples and reference sequences for assemblage A and B.
(XLS)

S5 Table Alignments of tpi sequences of samples and reference sequences for assemblage A and B.
(XLS)

S1 File Survey applied to guardian’s child. Social and environmental factors related to intestinal parasite infection, in English and Spanish.
(PDF)

S2 File PCR-RFLP analysis of *Giardia* samples. (PDF)
