# Supplementary material for: Intestinal parasitic infections and associated factors in children of three rural schools in Colombia. A cross-sectional study
Source: PLoS One. 2019 Jul 10;14(7):e0218681. doi: 10.1371/journal.pone.0218681 (PMC6619675; doi:10.1371/journal.pone.0218681)
Supplement: S1 Table — (PDF) [file pone.0218681.s001.pdf]

Table S1. Accession numbers obtained from Genbank for sequences used for *G. intestinalis* assemblage identification

	Gene marker		
**Assemblage**	**gdh**	**tpi**	**bg**
**AI**	L40509	L02120	AY258617
**AII**	AY178737	U57897	AY072723
**AII(A3)**	-	-	AY072724
**BIII**	AF069059	AF069561	AY072726
**BIV**	AY178738	AF069560	AY072728
**C**	U60982	AY228641	AY545646
**D**	U60986	DQ246216	AY545647
**E**	AY178740	KF891311	AY072729
**F**	AF069057	AF069558	AY647264
**G**	AY178745	EU781013	EU769221
