# Supplementary material for: Intestinal parasitic infections and associated factors in children of three rural schools in Colombia. A cross-sectional study
Source: PLoS One. 2019 Jul 10;14(7):e0218681. doi: 10.1371/journal.pone.0218681 (PMC6619675; doi:10.1371/journal.pone.0218681)
Supplement: S2 Table — (PDF) [file pone.0218681.s002.pdf]

Table S2. Associated factors with *Giardia intestinalis* infections in children of three rural schools in Colombia.

**Factor**	*Giardia* Positives n=38		*Giardia* Negatives n=59		Crude model			Multivariate model**		
	n	%	n	%	OR*	IC 95%	p	OR**	IC 95%	p
**Family size**										
≤ 4	12	31.58	19	32.20	1.0			1.0		
5 - 6	8	21.05	27	45.76	0.46	0.16 – 1.36	0.166	0.65	0.20 – 2.04	0.461
≥ 7	18	47.37	13	22.03	2.19	0.79 – 6.05	0.130	1.89	0.61 - 5.82	0.267
**Treatment of water for consumption**										
Boiled/chlorinated/filtrated	20	52.63	52	88.14	1.0			1.0		
None	18	47.37	7	11.86	6.68	2.42 – 18.42	**<0.001**	4.89	1.58 – 14.94	**0.006**
**Use of shoes**										
No	5	13.89	13	22.03	1.0			1.0		
Yes	31	86.11	46	77.97	0.57	0.18 – 1.76	0.330	0.86	0.22 – 3.24	0.826
**Raw fruit and vegetable consumption**										
No	5	13.89	16	27.12	1.0			1.0		
Yes	31	86.11	43	72.88	2.30	0.76 – 6.96	0.138	2.25	0.69 – 7.38	0.178
**History of intestinal parasites infections**										
No	13	34.12	22	37.29	1.0			1.0		
Yes	25	65.79	37	62.71	1.14	0.48 – 2.68	0.758	1.44	0.55 – 4.01	0.477
**History of diarrhea the last 15 days**										
No	34	89.47	50	84.75	1.0			1.0		
Yes	4	10.53	9	15.25	0.65	0.18 – 2.29	0.507	0.93	0.22 – 3.83	0.929
**History of abdominal pain the last 15 days**										
No	20	52.63	28	47.46	1.0			1.0		
Yes	18	47.37	31	52.54	0.81	0.35 – 1.83	0.619	0.93	0.36 – 2.39	0.881
**Members of the same household with diarrhea the last 15 days**										
No	33	86.84	48	81.36	1.0			1.0		
Yes	5	13.16	11	18.64	0.66	0.21 – 2.08	0.479	0.79	0.22 – 2.81	0.718

OR, odds ratio

*Estimated using logistic regression.

** Logistic regression adjusted by child's age, school, family income, crowding, material of construction of household and drinking water when the factor studied did not included one of those variables as independent variable
