# Supplementary material for: Intestinal parasitic infections and associated factors in children of three rural schools in Colombia. A cross-sectional study
Source: PLoS One. 2019 Jul 10;14(7):e0218681. doi: 10.1371/journal.pone.0218681 (PMC6619675; doi:10.1371/journal.pone.0218681)
Supplement: S1 File — Social and environmental factors related to intestinal parasite infection, in English and Spanish. (PDF) [file pone.0218681.s006.pdf]

UNIVERSIDAD EL BOSQUE

PROJECT: Study of intestinal parasites in rural schoolchildren from the municipality of Apulo (Cundinamarca)

Survey: Social and environmental factors related to intestinal parasite infection. Survey No.__________

1. School: ______________________________ 2. Date (dd/mm/yy): ___________________________

3. Parent or guardian's name:____

4. Parent or guardian's gender: Female ☐ Male ☐

5. Parent or guardian's age: _________ years 5.1. Date of birth (dd/mm/yy): ____________________

6. Relationship with the child: ____

7. Familiy's house location /place of residence: Rural ☐ Urban ☐ Neighborhood: ___________________

8. Phone number/Mobile number: ___________________________ ______________________________

9. Complete child's name: ____

SOCIO-DEMOGRAPHIC QUESTIONS

10: Parent or guardian's occupation:

a. Laborer ☐ b. Housewife ☐ c. Government employee ☐

d. Independent ☐ e. Housekeeper ☐ f. Non-Government employee ☐

g. Farmer ☐ h. Other. ____

11. Family income during the last month was:

a. Less than COP$ 500.000 ☐ b. Between COP$ 500.000 and COP$ 1.000.000 ☐

c. Between COP$ 1.000.000 and COP$ 1.500.000 ☐ d. More than COP$ 1.500.000 ☐

12. Family income was:

a. Not enough to cover the minimum expenses ☐ b. Just enough to cover the minimum expenses ☐

c. More than enough to cover the minimum expenses ☐

13: Parent or guardian's highest level of education:

a. None ☐ b. Unfinished primary school ☐ c. Primary school degree ☐

d. Unfinished high school ☐ e. High school degree ☐ f. Unfinished technical/college degree ☐

g. Technical/college degree ☐ h: Other: ______________________________

14: Do you identify yourself as?:

a. Without any particular ethnicity ☐ b. Indigenous ☐ c. Afro-Colombian ☐

d: Other: ______________________________

15: What kind of housing do you live in?:

a. House ☐ b. Apartment ☐ c. Room ☐ d. Other: ______________

16: How many people live in there?:

____

17: How many rooms does it have (do not include kitchens, bathrooms or parking spaces)?:

____

18: How many families prepare meals in the house?:

____

19: Someone in this household actively participates in any community benefit organization?:

a. NO ☐ b. YES ☐ c. Which organization: ______________________________

20: Does any member of the family receive welfare subsidies?:

a. NO ☐ b. YES ☐ c. Which one: ______________________________

21: Where and with whom does the child stay when she/he are not in school?:

a. With her/his mother/father at home ☐ b. With her/his mother/father at work ☐

c. With a nanny at home ☐ d. With a relative over 18 years of age (legal age) ☐

e. With a relative under 18 years of age ☐ f. At home, alone

g. Other: ______________________________

22. Did any family member skip a meal (breakfast, lunch or dinner) one or more days of the last week due to lack of money?:

a.YES ☐ b. NO ☐

INFRASTRUCTURE, WATER, SANITATION AND HYGIENE QUESTIONS

23: The house is built with?:

a. Cement/brick or prefabricated house ☐ b. Stone ☐

c. Adobe/Giant bamboo ☐ d. Other: ____

24: What material is the floor made of?:

a. Soil ☐ b. Cement/tiles ☐ c. Other: ____

25: Public services in the house:

a. Energy ☐ b. Home gas ☐ c. Gas (cylinder) ☐

d. Aqueduct system ☐ e. Sewerage system ☐ f. Phone ☐

g. Waste collection ☐ h. None ☐

26: Where does the water for human consumption come from?:

a. Rural aqueduct ☐ b. Community aqueduct ☐ c. Rainwater ☐

d. Water tank truck ☐ e. Well or tank with lid ☐ f. Well or tank without lid ☐

g. Protected river/stream ☐ h. Unprotected river/stream ☐ i. Bottled water ☐

j. Other: ____

27: How often does the family have access to water?

a. Daily ☐ b. 2 or 3 times per week ☐ c. Weekly ☐

d. Biweekly ☐ e. Monthly ☐ f. Other: ________________________

27.1: Do you have water service all day?

a. YES ☐ b. NO ☐ c. This does not apply ☐

28: Do you do any treatment to purify the water for human consumption?

a. Boil ☐ b. Filter ☐ c. Treat with hypochlorite ☐

d. Decant ☐ e. Sun disinfection ☐ f. None: ________________________

29: Where do the family members urinate and defecate?

a. Toilet connected to the sewer system ☐ b. Toilet connected to septic tank ☐

c. Nonwater carriage toilet ☐ d. Latrine….☐

e. Latrine with ventilation ☐ f. On the field ☐

g. Other: ____

29.1: If you answered “a”, “b”, “c”, “d” or “e” in the previous question, where are those installations?

a. Indoors ☐ b. House surroundings ☐ c. Outside of property ☐

d. This does not apply ☐ e. Other: ____

29.2. If you answered “b” in question 29, when was done the last maintenance (pumped out) of the septic tank?

a. Years _________ Months ___________ b. You do not know ☐

c. Never ☐ d. This does not apply ☐

30. How does the solid waste is disposed?

a. They are delivered to the waste company ☐ b. They are buried ☐ c. They are burned ☐

d. They are thrown into field ☐ e. They are thrown into rivers ☐ f. Other. ________________

30.1 If solid waste is collected, how often is done the collection?

a. Every day ☐ b. Twice per week ☐ c. Weekly ☐

d. Biweekly ☐ e. Monthly ☐ f. Other. ________________

31. Are there animals living or continuously entering the house? Which?

a. Dogs ☐ b. Cats ☐ c. Hens ☐

d. Pigs ☐ e. None ☐ f. Other. ________________

32. Do family members walk without shoes inside or outside the house?

a. YES ☐ b. NO ☐

d. Pigs ☐ e. None ☐ f. Other. ________________

33. Do family members eat raw vegetables?

a. YES ☐ b. NO ☐

34. Are vegetables and fruits washed before they are consumed?

a. YES ☐ b. NO ☐

35. How vegetables and fruits are washed?

a. With raw water ☐ b. With boiled water ☐ c. With treated water (hypochlorite) ☐

d. Other. ________________

36. At home, do family members wash their hands before preparing meals?

a. YES ☐ b. NO ☐

37. At home, do family members wash their hands before eating?

a. YES ☐ b. NO ☐

38. At home, do family members wash their hands after going to the bathroom?

a. YES ☐ b. NO ☐

39. At home, is water collected in tanks?

a. YES ☐ b. NO ☐

40. How often the tanks are cleaned?

a. Semiannually ☐ b. Annually ☐ c. Less frequent than annually ☐

d. Never ☐

40.1. When was the last tank maintenance (wash)?

a. Years _________ Months ___________ b. You do not know ☐

c. Never ☐ d. This does not apply ☐

Name and Signature of the survey applicator	Date (dd/mm/yy): ____________________

UNIVERSIDAD EL BOSQUE

Estudio de parasitosis intestinales en niños de escuelas rurales del municipio de Apulo (Cundinamarca)

N° encuesta ☐

ENCUESTA: Factores de Riesgo Sociales y Ambientales Relacionados con Parasitosis Intestinales

1. Escuela ____________________

2. Fecha de la encuesta (dia/mes/año) ____________

DATOS DEL ENTREVISTADO

3. Nombre del adulto (padre/acudiente): ____________________

4. Sexo del acudiente: Masculino ☐ Femenino ☐

5. Edad acudiente ☐ Años 5.1. Fecha de Nacimiento acudiente (día/mes/año) ☐ ☐ ☐

6. Parentesco con el estudiante ____________________

7. Ubicación de la vivienda: Rural ☐ Urbano ☐ Barrio: ____________

8. Teléfono fijo y/o celular de contacto: ____________

9. Nombre y apellidos estudiante ____________________

ASPECTOS SOCIODEMOGRÁFICOS

10. Usted a qué se dedica: ***(No leer opciones)***
- a. Jornalero o peón ☐
- b. Ama de casa ☐
- c. Obrero o empleado del gobierno ☐
- d. Independiente ☐
- e. Empleado doméstico ☐
- f. Obrero o empleado de empresa particular ☐
- g. Trabajador de finca ☐
- h. Otro ¿Cuál? ☐ ____________

11. El mes anterior los ingresos familiares fueron:
- a. Menos de $500.000 ☐
- b. Entre $500.000 y $1.000.000 ☐
- c. Entre $1.000.000 y $1.500.000 ☐
- d. Más de $1.500.000 ☐

12. Los ingresos de su hogar:
- a. No alcanzan para cubrir los gastos mínimos ☐
- b. Sólo alcanzan para cubrir los gastos mínimos ☐
- c. Cubren más que los gastos mínimos ☐

13. ¿Cuál es su nivel educativo? ***(Una sola opción de respuesta)***
- a. Ninguno ☐
- b. Primaria incompleta ☐
- c. Primaria completa ☐
- d. Secundaria incompleta ☐
- e. Secundaria completa ☐
- f. Técnico o universitario incompleto ☐
- g. Técnico o universitario completo ☐
- h. Otro ¿Cuál? ☐ ____________

14. ¿De acuerdo con su cultura, pueblo o rasgos físicos usted es o se reconoce como: ***(Leer opciones)***
- a. Sin pertenencia étnica ☐
- b. Indigena ☐
- c. Negro,mulato, Afrocolombiano o afrodescendiente ☐
- d. Otro ¿Cuál? ☐ ____________

15. En qué tipo de vivienda reside ***(No leer las opciones)***
- a. Casa ☐
- b. Apartamento ☐
- c.Habitación/Cuarto ☐
- d. Otro ¿Cuál? ☐ ____________

16. ¿Cuántas personas habitan la vivienda? ________

17 ¿Cuántos cuartos (incluidos sala y comedor) tiene la vivienda? ________
(Excluya cocinas, baños, garajes y cuartos destinados a negocio)

18. ¿Cuántos grupos de personas (hogares) preparan los alimentos por separado en esta vivienda? ________

19. ¿Alguien de este hogar PARTICIPA ACTIVAMENTE en alguna organización de beneficio comunitario?
- a. Si ☐ 19.1 ¿Cuál organización?
- b. No ☐ ________________________________

20. Algún miembro del hogar se encuentra inscrito en algún programa de beneficio social?
- a. Si ☐ 20.1 ¿Cuál programa? ________________________________
- b. No ☐

21. ¿Dónde o con quién permanece el(los) estudiante(s) durante la mayor parte del tiempo mientras no está en la escuela entre semana?
- a. Con su padre o madre en la casa ☐
- b. Con su padre o madre en el trabajo ☐
- c. Con empleada o niñera en la casa ☐
- d. Al cuidado de un pariente de 18 años o más ☐
- e. Al cuidado de un pariente menor de 18 años ☐
- f. En casa solo ☐
- g. Otro ¿Cuál? ________________

22. ¿Por falta de dinero, algún miembro del hogar no consumió ninguna de las tres comidas (desayuno, almuerzo, comida), durante uno o más días de la semana pasada?
- a. Si ☐
- b. No ☐

CONDICIONES DE INFRAESTRUCTURA, AGUA, SANEAMIENTO E HIGIENE

23. La vivienda esta construida con:
- a. Cemento/Ladrillo, Prefabricada ☐
- b. Piedra ☐
- c. Bahareque / Guadua ☐
- d. Otro ¿Cuál? ☐ ________________

24. El piso de la vivienda esta hecho de:
- a. Tierra ☐
- b. Cemento/Baldosa ☐
- c. Otro ¿Cuál? ☐ ________________

25. ¿Con cuáles servicios públicos cuenta la vivienda?
- a. Energía eléctrica ☐
- b. Gas natural conectado ☐
- c. Gas cilindro (pipeta) ☐
- d. Acueducto ☐
- e. Alcantarillado ☐
- f. Teléfono fijo ☐
- g. Recolección de basuras ☐
- h. Ninguno ☐

26. ¿De dónde obtiene el agua para consumo (beber o preparar alimentos)?
- a. Acueducto municipal ☐
- b. Acueducto veredal ☐
- c. Agua lluvia ☐
- d. Carro tanque ☐
- e. Pozo o aljibe con tapa ☐
- f. Pozo o aljibe sin tapa ☐
- g. Río, quebrada, nacedero con protección ☐
- h. Río, quebrada, nacedero sin protección ☐
- i. Agua embotellada o en bolsa ☐
- j. Otro ¿Cuál? ☐ ________________

27. ¿Cada cuánto llega el agua a la vivienda?
- a. Diariamente ☐
- b. Dos o 3 veces por semana ☐
- c. Semanal ☐
- d. Quincenal ☐
- e. Mensual ☐
- f. Otro ¿Cuál? ☐ ________________

27.1 ¿Tiene servicio de agua todo el día?
- a. Si ☐
- b. No ☐
- c. No Aplica ☐

28. ¿Realiza algún procedimeinto/tratamiento interno para tratar o purificar el agua de consumo?
- a. Hervir ☐
- b. Filtrar ☐
- c. Clorar ☐
- d. Dejar Reposar ☐
- e Desinfección Solar ☐
- f. Ninguno ☐
- g. Otro ¿Cuál? ☐ ____________

29. ¿Dónde hacen las necesidades (orinan y defecan) los miembros del hogar?
- a. Sanitario con conexión a alcantarillado ☐
- b. Sanitario con conexión a pozo séptico ☐
- c. Sanitario sin conexión a alcantarillado ni a pozo séptico ☐
- d. Letrina ☐
- e. Letrina mejorada con ventilación ☐
- f. Al aire libre ☐
- g. Otro ¿Cuál? ☐ ____________

29.1. Si en pregunta "29" respondio de "a - e": ¿Donde se encuentra(n) esta(s) instalación(es)?
- a. En el intradomicilio ☐
- b. En el peridomicilio ☐
- c. Fuera de la vivienda ☐
- d. No aplica ☐
- e. Otro ¿Cuál? ☐ ____________

29.2. Si en pregunta "29" respondio "b" (sanitario con conexión a pozo septico):
¿Cuándo fue la última vez que se hizo mantenimiento al pozo séptico (reparar y vaciar el contenido del pozo)?
- a. Meses _______ / años ________
- b. No sabe ☐
- c. Nunca ☐
- d. No aplica ☐

30. ¿Cómo son dispuestas las basuras (residuos sólidos) que se generan en la vivienda?
- a. Se entregan al servicio de aseo ☐
- b. La entierran ☐
- c. La queman ☐
- d. La tiran a campo abierto ☐
- e. La tiran a un río, caño, quebrada ☐
- f. Otro ¿Cuál? ☐ ____________

30.1. Si son recogidos, ¿Cada cuánto son recogidos los residuos sólidos?
- a. Todos los días ☐
- b. 2 veces por semana ☐
- c. Semanal ☐
- d. Quincenal ☐
- e. Mensual ☐
- f. Otro ¿Cuál? ☐ ____________

31. ¿Qué animales viven o entran a la vivienda?
- a. Perros ☐
- b. Gatos ☐
- c. Gallinas ☐
- d. Cerdos ☐
- e. No entran ☐
- f. Otro ¿Cuál? ☐ ____________

32. Los miembros de su hogar caminan descalzos por la vivienda (intra y peridomicilio)?
- a. Si ☐
- b. No ☐

33. ¿En su hogar comen verduras crudas?
- a. Si ☐
- b. No ☐

34. ¿Se lavan las frutas y hortalizas antes de su preparación?
- a. Si ☐
- b. No ☐

35. ¿Cómo se lavan las frutas y verduras antes de su preparación?
- a. Con agua cruda ☐
- b. Con agua hervida ☐
- c. Con agua con cloro /sal ☐
- f. Otro ¿Cuál? ☐ ____________

36. En su hogar se lavan las manos antes de preparar alimentos
- a. Si ☐
- b. No ☐

37. ¿En la casa se lavan las manos antes de comer?
- a. Si ☐
- b. No ☐

38. ¿En la casa se lavan las manos después de ir al baño?

a. Si ☐ b. No ☐

39. En su vivienda ¿Almacena el agua en tanques?

a. Si ☐ b. No ☐

40. Frecuencia de lavado de los tanques

a. Semestral ☐ c. Mayor a anual ☐ e. No aplica ☐

b. Anual ☐ d. Nunca ☐

40.1 Cuando fue la ultima vez que lavó sus tanques de agua:

a. Meses _______ / años ________ c. Nunca ☐

b. No sabe ☐ d. No aplica ☐

**Nombre y Firma del Encuestador**	**Fecha (dd/mm/aaa)**
	______ / ______ / ______________
