# Supplementary material for: Intestinal parasitic infections and associated factors in children of three rural schools in Colombia. A cross-sectional study
Source: PLoS One. 2019 Jul 10;14(7):e0218681. doi: 10.1371/journal.pone.0218681 (PMC6619675; doi:10.1371/journal.pone.0218681)
Supplement: S2 File — (PDF) [file pone.0218681.s007.pdf]

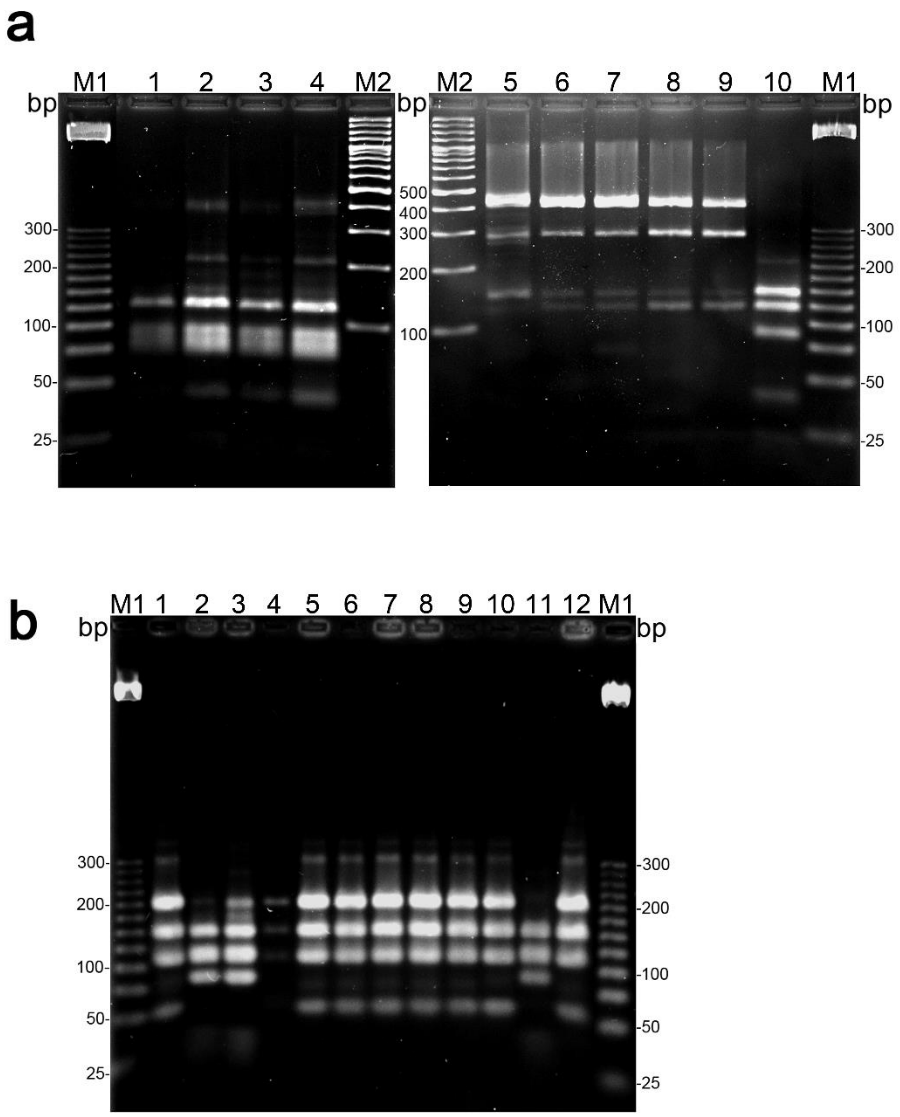

S2 File. PCR-RFLP analysis of *Giardia* samples

Agarose Super Fine Resolution gels (2%) stained with ethidium bromide showing electrophoretic separation of PCR products after digestion with restriction enzymes.

Panel a: nested *gdh* PCR product (432 bp) digested with NlaIV. Assemblage AII: lanes 1 to 4; samples T11, T12, T14 and T21, respectively. Assemblage B: lanes 5 to 9; samples P16, P21, P24, P28 and P29, respectively. Assemblage AI: lane 10; sample P23. M1: Molecular Weight Marker 25 bp (Promega). M2: Molecular Weight Marker 100 bp (Thermo).

Panel b: nested *B-giardin* PCR product (511 bp) digested with HaeIII. Assemblage A: lanes 1, and 4 to 10; samples P16, T31, T11, T12, T14, T21, T24, T34, respectively. Lane 12: DNA control, WB strain. Assemblage B: lanes 2, 3 and 11; samples P21, P24 and T37, respectively. M1: Molecular Weight Marker 25 bp (Promega).
